# Carbamazepine‐Induced Stevens‐Johnson Syndrome/Toxic Epidermal Necrolysis Overlap With Severe Ocular and Oral Involvement: A Pediatric Case Report From Uganda

**DOI:** 10.1002/ccr3.73474

**Published:** 2026-09-03

**Authors:** Abdisalam Ahmed Sandeyl, Abdisamad Guled Hersi, Zakarie Abdullahi Hussein, Mohamed Farah Ismail, Abdullahi Ali Bulle, Mamoun Nasr

**Affiliations:** ^1^ Internal Medicine Department Kampala International University Western Campus Bushenyi Uganda; ^2^ Opthalmology Department Kampala International University Western Campus Bushenyi Uganda; ^3^ Surgery Department Kampala International University Western Campus Bushenyi Uganda; ^4^ Dermatology Department Kampala International University Western Campus Bushenyi Uganda

**Keywords:** carbamazepine, ocular involvement, pediatric, SJS/TEN overlap, Steven‐Johnson syndrome, toxic epidermal necrolysis

## Abstract

We report a case of carbamazepine‐induced Stevens‐Johnson syndrome/toxic epidermal necrolysis (SJS/TEN) overlap in an 11‐year‐old Ugandan boy who presented with fever, facial swelling, ocular redness with photophobia, painful oral ulceration, and widespread epidermal detachment involving more than 10% of the body surface area. Symptoms developed 10 days after initiation of carbamazepine for newly diagnosed epilepsy. Examination revealed extensive mucocutaneous involvement characterized by bilateral eyelid edema, conjunctival inflammation, perioral hemorrhagic crusting, erosive oral ulcers, and a positive Nikolsky sign. A clinical diagnosis of carbamazepine‐induced SJS/TEN overlap was established based on the temporal relationship to drug exposure and characteristic clinical findings. Immediate withdrawal of carbamazepine and multidisciplinary supportive care resulted in marked clinical improvement. This case highlights the importance of early recognition, prompt withdrawal of the offending drug, and multidisciplinary supportive care in improving outcomes for pediatric SJS/TEN, particularly in resource‐limited settings.

## Key Clinical Message

Carbamazepine is a well‐recognized high‐risk trigger of SJS/TEN in children and may present with rapidly progressive severe mucocutaneous and ocular involvement. Early recognition, immediate withdrawal of the offending drug, and prompt multidisciplinary supportive care are essential to improve outcomes, particularly in resource‐limited settings.

**Abbreviations**
DRESS: Drug Reaction with Eosinophilia and Systemic Symptoms
SCORTEN: Severity‐of‐Illness Score for Toxic Epidermal Necrolysis
SJS: Stevens‐Johnson Syndrome
SJS/TEN: Stevens‐Johnson Syndrome/Toxic Epidermal Necrolysis
SSSS: Staphylococcal Scalded Skin Syndrome
TEN: Toxic Epidermal Necrolysis

## 1 Introduction

Stevens‐Johnson syndrome (SJS) and toxic epidermal necrolysis (TEN) are rare but life‐threatening mucocutaneous hypersensitivity reactions characterized by widespread keratinocyte apoptosis and epidermal detachment. SJS/TEN overlap is defined by epidermal detachment involving 10%–30% of the body surface area and typically presents with a prodrome of fever and malaise followed by rapidly progressive blistering eruptions and mucosal involvement [1]. Early recognition and prompt withdrawal of the offending agent are critical to reducing morbidity and mortality [2].

The global incidence of SJS/TEN is estimated at 1–5 cases per million annually, with mortality ranging from 5%–10% in SJS to 30% in TEN; patients with SJS/TEN overlap have an intermediate mortality risk. Management is largely supportive and often requires multidisciplinary care, particularly in cases with significant ocular involvement [3]. In sub‐Saharan Africa, the burden of SJS/TEN may be influenced by exposure to high‐risk medications and HIV infection, although epidemiological data remain limited [4]. Among implicated drugs, carbamazepine is a well‐recognized trigger of SJS/TEN and remains an important cause of severe cutaneous adverse reactions in children [5].

We report an 11‐year‐old Ugandan boy who developed carbamazepine‐induced SJS/TEN overlap with severe ocular and oral involvement, highlighting the challenges of diagnosing and managing severe cutaneous adverse drug reactions in resource‐limited settings and underscoring the importance of early drug withdrawal and multidisciplinary care.

## 2 Case Presentation

### 2.1 Case History and Examination

An 11‐year‐old boy weighing 32 kg was admitted to Kisoro District Hospital with a five‐day history of high‐grade fever, progressive facial swelling, painful red eyes with photophobia, widespread epidermal detachment, and painful oral ulceration. He had been diagnosed with epilepsy approximately 2 weeks prior and initiated on carbamazepine 200 mg twice daily. Symptoms developed approximately 10 days after carbamazepine initiation and rapidly progressed to involve the skin, oral mucosa, and eyes. Oral intake was significantly reduced due to painful oral lesions. There was no history of prior drug allergies, chronic illness, or family history of severe cutaneous adverse drug reactions.

On examination, the patient appeared acutely ill, irritable, and moderately dehydrated. Vital signs were: temperature 38.9°C, heart rate 121 beats/min, respiratory rate 24 breaths/min, blood pressure 102/64 mmHg, and oxygen saturation 96% on room air.

**FIGURE 1:**
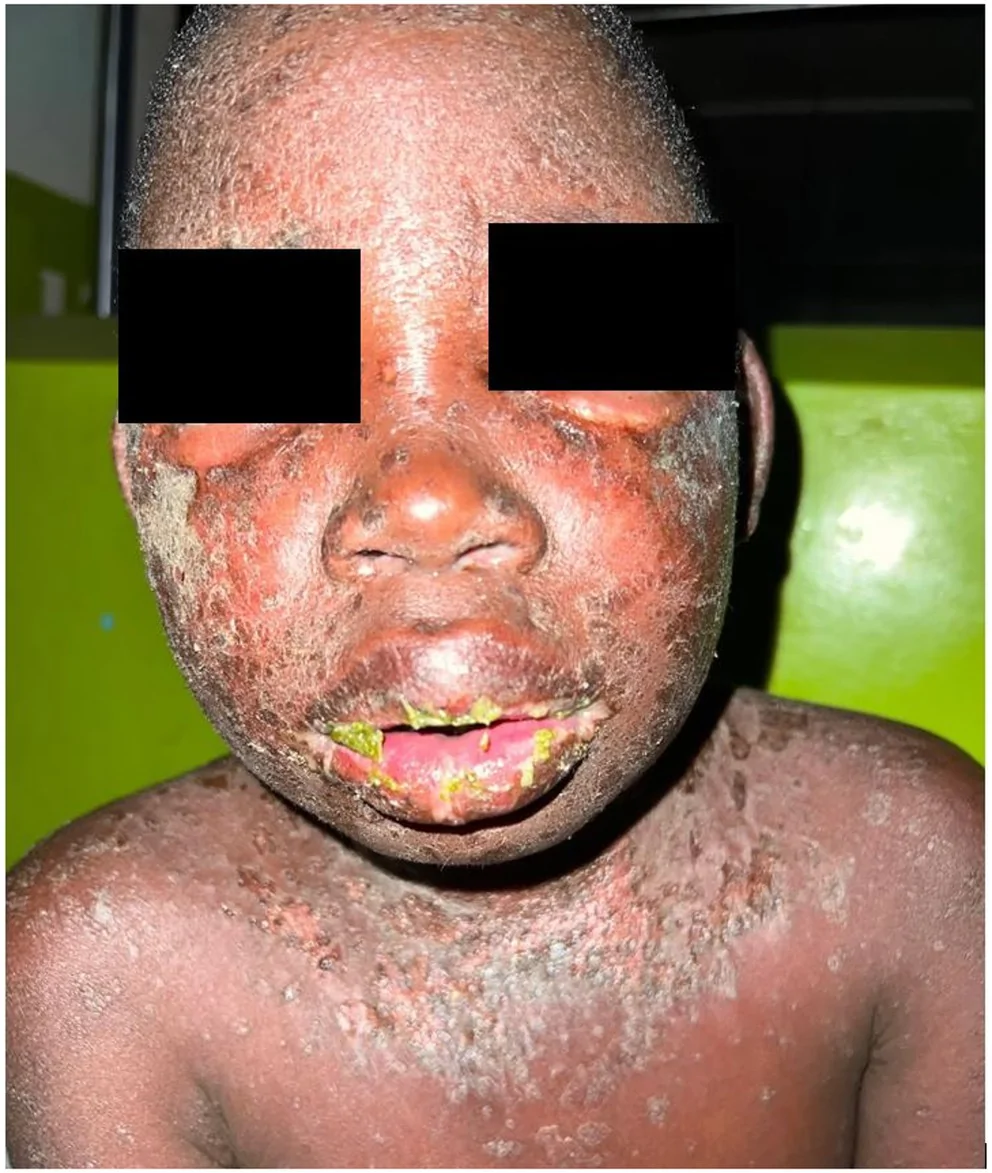
Facial and Oral Involvement in SJS/TEN Overlap. Widespread maculopapular eruption with epidermal detachment involving the face and neck, associated with bilateral eyelid edema, perioral crusting, and hemorrhagic oral erosions.

**FIGURE 2:**
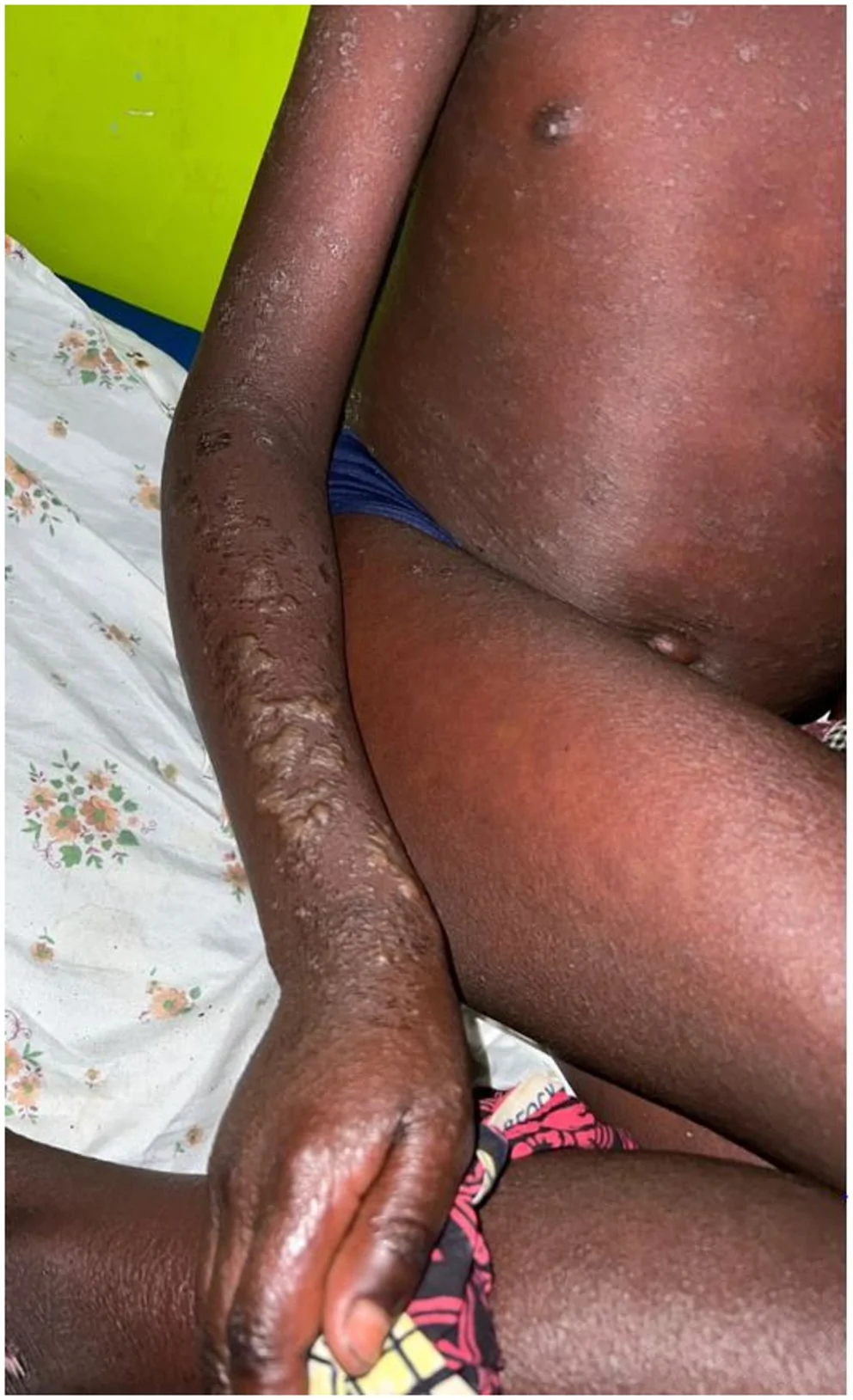
Cutaneous Blistering and Desquamation in SJS/TEN Overlap. Extensive blistering with epidermal detachment of the upper limb, with areas of healing erosions on the chest.

Dermatologic examination revealed widespread erythematous macules with blister formation and epidermal detachment involving > 10% of body surface area, predominantly affecting the face, neck, trunk, and extremities. There was extensive perioral hemorrhagic crusting with erosive oral ulcers, bilateral conjunctival inflammation with mucopurulent discharge, eyelid edema, and marked photophobia (Figure 1). The limbs demonstrated extensive epidermal detachment with erosions and early re‐epithelialization in some areas (Figure 2). Nikolsky sign was positive.

### 2.2 Investigations and Differential Diagnosis

Laboratory investigations showed mild leukocytosis with a white blood cell count of 12 × 10^9^/L (reference range: 4–11 × 10^9^/L). Liver and renal function tests, serum glucose, and bicarbonate levels were within normal limits. Skin biopsy and HLA‐B*15:02 testing were unavailable due to resource limitations. Severity‐of‐illness score for toxic epidermal necrolysis (SCORTEN) was 2, based on heart rate > 120 beats/min and epidermal detachment involving > 10% of body surface area, indicating an intermediate predicted mortality risk.

Based on the temporal relationship with carbamazepine initiation, characteristic mucosal and cutaneous findings, and extent of epidermal detachment, a clinical diagnosis of carbamazepine‐induced Stevens‐Johnson syndrome/toxic epidermal necrolysis (SJS/TEN) overlap was established.

Differential diagnoses included erythema multiforme major, staphylococcal scalded skin syndrome (SSSS), drug reaction with eosinophilia and systemic symptoms (DRESS), and generalized bullous fixed drug eruption. Erythema multiforme major was considered less likely due to the rapid progression, extensive epidermal detachment, and prominent mucosal involvement. SSSS was considered unlikely given the patient's age and the presence of significant mucosal involvement, which is typically absent in SSSS. DRESS syndrome was less favored due to the absence of eosinophilia, systemic organ involvement, and the relatively short latency period following carbamazepine initiation. Generalized bullous fixed drug eruption was considered less likely due to the extensive mucosal involvement and absence of the well‐demarcated recurrent plaques typical of fixed drug eruptions. Carbamazepine was the only recently introduced medication, strongly supporting carbamazepine‐induced SJS/TEN overlap as the underlying etiology.

### 2.3 Treatment and Outcome

Management included immediate discontinuation of carbamazepine, intravenous fluid resuscitation with normal saline and 5% dextrose, intravenous paracetamol for pain and fever control, empirical intravenous ceftriaxone, topical tetracycline and dexamethasone eye drops under ophthalmologic guidance for severe ocular involvement, and petroleum jelly‐based skin emollients. Nutritional support was provided via nasogastric feeding due to painful oral mucosal involvement and poor oral intake. Following clinical stabilization, carbamazepine was replaced with oral levetiracetam 250 mg twice daily for seizure control.

After 10 days of treatment, the patient demonstrated marked clinical improvement, with progressive re‐epithelialization of skin lesions, resolution of oral ulcers, and resumption of oral feeding. Ocular symptoms also improved, although mild photophobia persisted. He was discharged in stable condition with planned outpatient follow‐up at Mbarara Regional Referral Hospital for specialist ophthalmologic and neurologic review. The clinical course, interventions, and outcomes are summarized in Table 1.

**TABLE 1:** Clinical course and management of carbamazepine‐Induced SJS/TEN overlap in an 11‐year‐old child. Summary of the patient's clinical course, therapeutic interventions, and outcomes during hospitalization.

Day	Event/intervention	Clinical findings/outcome
14 prior	Carbamazepine initiated (200 mg twice daily)	Started for newly diagnosed epilepsy
5 prior	Onset of symptoms	Fever, facial swelling, conjunctival redness, oral ulcers; rapid progression with photophobia and reduced oral intake
0	Admission to Kisoro District Hospital	Dermatologic examination showed > 10% BSA involvement with positive Nikolsky sign; mild leukocytosis; liver and renal function tests normal; SCORTEN = 2
0	Carbamazepine discontinued	Immediate withdrawal of suspected offending drug
0–10	Supportive management	Intravenous fluids, empirical antimicrobial therapy, analgesia, topical ocular treatment, petroleum jelly‐based emollient, and nasogastric feeding; gradual clinical improvement noted
10	Antiepileptic switched	Levetiracetam initiated (250 mg twice daily) after stabilization
10–13	Continued inpatient care	Progressive healing of skin lesions; resolution of oral ulcers; improvement in ocular symptoms with mild residual photophobia
13	Discharge	Discharged with referral to Mbarara Regional Referral Hospital for specialist ophthalmology and neurology follow‐up

Abbreviations: BSA, Body surface area; SCORTEN, Severity‐of‐Illness Score for Toxic Epidermal Necrolysis.

## 3 Discussion

### 3.1 Etiology and Risk Factors

Medications are the predominant triggers of pediatric SJS/TEN, with antiepileptic drugs among the most frequently implicated classes worldwide [5]. Carbamazepine is a well‐recognized high‐risk agent, with symptom onset typically occurring within 2–8 weeks of treatment initiation [6]. In the present case, symptoms developed approximately 10 days after carbamazepine exposure, consistent with the expected latency period for drug‐induced SJS/TEN.

Pharmacogenetic screening for HLA‐B*15:02 has significantly reduced carbamazepine‐associated SJS/TEN in East and Southeast Asian populations; however, this allele appears to be uncommon in African populations [7]. This suggests that additional genetic, environmental, or population‐specific susceptibility factors may contribute to the development of SJS/TEN in sub‐Saharan Africa.

### 3.2 Ocular Involvement

Ocular involvement is a major cause of morbidity in SJS/TEN and significantly impacts long‐term quality of life [8]. Early manifestations, including conjunctivitis, eyelid edema, and photophobia, as observed in this patient, warrant prompt ophthalmologic evaluation. Early treatment with topical antibiotics, corticosteroids, and intensive lubrication during the acute phase may reduce the risk of chronic sequelae such as symblepharon, corneal scarring, and permanent visual impairment [9]. In this case, early ophthalmologic intervention was associated with a favorable outcome, with only mild residual photophobia at discharge.

### 3.3 Supportive Care and Management

Supportive care remains the cornerstone of SJS/TEN management and is strongly associated with improved outcomes compared with specific immunomodulatory therapies. Core principles include immediate withdrawal of the offending drug, meticulous fluid and electrolyte management, effective pain control, nutritional support, and close monitoring for infection [3]. Empirical antimicrobial therapy is often initiated in resource‐limited settings in extensive disease due to skin barrier disruption and limited microbiological diagnostics, as described in similar contexts [4].

In this patient, early supportive management: including fluid resuscitation, pain control, topical ocular therapy, and nutritional support was associated with favorable clinical recovery despite an intermediate predicted mortality risk (SCORTEN = 2) [10].

### 3.4 Challenges in Resource‐Limited Settings

In this setting, limited access to confirmatory diagnostic tools, including skin biopsy and HLA‐B*15:02 testing, constrained etiological confirmation. In addition, limited availability of specialized ophthalmologic services posed challenges for comprehensive ocular assessment. These constraints may contribute to delayed diagnosis and early intervention in severe cutaneous adverse drug reactions such as SJS/TEN, with potential implications for morbidity and long‐term outcomes. Strengthening early referral pathways, improving access to multidisciplinary care, and enhancing clinician awareness are critical to improving outcomes in pediatric SJS/TEN in sub‐Saharan Africa [4].

### 3.5 Clinical Implications

Clinicians should maintain a high index of suspicion for SJS/TEN in children who develop acute mucocutaneous symptoms shortly after initiation of high‐risk medications, including carbamazepine. Early recognition, immediate discontinuation of the offending drug, and timely initiation of supportive care are critical to improving outcomes, even in settings without access to advanced diagnostics or pharmacogenetic testing.

## 4 Conclusion

Carbamazepine‐induced Stevens‐Johnson syndrome/toxic epidermal necrolysis (SJS/TEN) overlap is a rare but potentially life‐threatening adverse drug reaction in children. Early recognition and immediate withdrawal of the offending drug remain the most critical steps in management, alongside supportive care. This case highlights the importance of timely multidisciplinary intervention in improving outcomes in a resource‐limited setting. Continued surveillance and further research are needed to improve understanding and management of pediatric drug‐induced SJS/TEN in Uganda.

## Author Contributions

**Abdisalam Ahmed Sandeyl:** conceptualization, investigation, writing – original draft, writing – review and editing, project administration. **Abdisamad Guled Hersi:** conceptualization, investigation, writing – review and editing. **Zakarie Abdullahi Hussein:** investigation, data curation. **Mohamed Farah Ismail:** investigation, resources. **Abdullahi Ali Bulle:** investigation, writing – review and editing. **Mamoun Nasr:** supervision, resources, writing – review and editing.

## Funding

The authors have nothing to report.

## Consent

Written informed consent was obtained from the patient's parent for publication of this case report and any accompanying images.

## Conflicts of Interest

The authors declare no conflicts of interest.

## Data Availability

The data that support the findings of this study are available from the corresponding author upon reasonable request.
